# Long-term prognosis of breast cancer detected by mammography screening or other methods

**DOI:** 10.1186/bcr3080

**Published:** 2011-12-28

**Authors:** Tiina Lehtimäki, Mikael Lundin, Nina Linder, Harri Sihto, Kaija Holli, Taina Turpeenniemi-Hujanen, Vesa Kataja, Jorma Isola, Heikki Joensuu, Johan Lundin

**Affiliations:** 1 Institute for Molecular Medicine Finland (FIMM), University of Helsinki, Biomedicum Helsinki 2U, Tukholmankatu 8, PO Box 20, FI-00014 Helsinki, Finland; 2 Molecular Cancer Biology Program, Biomedicum Helsinki, University of Helsinki, Tukholmankatu 8, PO Box 20, FI-00014, Helsinki, Finland; 3 Departments of Palliative Medicine and Oncology, Tampere University Hospital and University of Tampere, Teiskontie 35, PO Box 2000, FI-33521,Tampere, Finland; 4 Department of Oncology and Hematology, Oulu University Central Hospital, Kajaanintie 50, PO Box 20, FI-90029, Oulu, Finland; 5 Cancer Center, Kuopio University Central Hospital, Puijonlaaksontie 2, PO Box 1777, FI-70211, Kuopio, Finland; 6 Institute of Medical Technology, University of Tampere and Tampere University Hospital, Biokatu 8-12, PO Box 2000, FI-33520,Tampere, Finland; 7 Department of Oncology, Helsinki University Central Hospital, Haartmaninkatu 4, PO Box 180, FI-00029, Helsinki, Finland; 8 Division of Global Health, Karolinska Institutet, SE-17177, Stockholm, Sweden

**Keywords:** screening, mammography, prognosis, survival analysis

## Abstract

**Introduction:**

Previous studies of breast cancer have shown that patients whose tumors are detected by mammography screening have a more favorable survival. Little is known, however, about the long-term prognostic impact of screen detection. The purpose of the current study was to compare breast cancer-specific long-term survival of patients whose tumors were detected in mammography screening compared with those whose tumors were detected by other methods.

**Methods:**

Breast cancer patients diagnosed within five specified geographical areas in Finland in 1991 and 1992 were identified (*N *= 2,936). Detailed clinical, treatment and outcome data, as well as tissue samples, were collected. Women with *in situ *carcinoma, distant metastases at the time of primary diagnosis and women who were not treated surgically were excluded. The main analyses were performed after excluding patients with other malignancy or contralateral breast cancer, followed by sensitivity analyses with different exclusion criteria. Median follow-up time was 15.4 years. Univariate and multivariate analyses of breast cancer-specific survival were performed.

**Results:**

Of patients included in the main analyses (*n *= 1,884), 22% (*n *= 408) of cancers were screen-detected and 78% (*n *= 1,476) were detected by other methods. Breast cancer-specific 15-year survival was 86% for patients with screen-detected cancer and 66% for patients diagnosed using other methods (*P *< 0.0001, HR = 2.91). Similar differences in survival were observed in women at screening age (50 to 69 years), as well as in clinically important subgroups, such as patients with small tumors (≤ 1 cm in diameter) and without nodal involvement (N0). Women with breast cancer diagnosed on the basis of screening mammography had a more favorable prognosis than those diagnosed outside screening programs, following adjustments according to patient age, tumor size, axillary lymph node status, histological grade and hormone receptor status. Significant differences in the risk of having future contralateral breast cancer according to method of detection were not observed.

**Conclusions:**

Breast cancer detected by mammography screening is an independent prognostic factor in breast cancer and is associated with a more favorable survival rate as well as in long-term follow-up.

## Introduction

Breast cancers detected by screening mammography have more favorable prognostic characteristics than cancers detected by other methods [1-9]. The tumors are smaller, are more often well-differentiated, show less spread to regional lymph nodes and have a lower proliferation index [1,2,10-16]. In addition, a large proportion of screen-detected tumors are of luminal type A, and relatively few are of the human epidermal growth factor receptor 2-positive/estrogen receptor-negative (HER2+/ER-) molecular subtype [9]. Studies have shown the method of detection to be an independent prognostic factor, even after adjustment for a series of established prognostic variables [9,17,18].

In a recently published study of 2,592 Dutch breast cancer patients, screen detection was reported to be independently associated with better breast cancer-specific and overall survival after a median follow-up of 11 years [18]. We observed similar results in our previous study [17] in which patients with screen-detected breast cancer had significantly better distant disease-free survival than patients whose tumors were detected by other methods. Adjustments for several known prognostic factors were made in both studies, which reduced the effect of lead time and length biases. Screening as a method of detection has been considered an independent prognostic factor in several other studies [3,12,19-21].

Most breast cancer recurrences occur 2 to 3 years after diagnosis [22], but the disease recurs even 15 to 25 years after diagnosis in some patients [23-25]. A time-dependency has been reported for prognostic factors such as ER status, tumor size, lymph node status and tumor grade, whereby the prognostic value may decrease with time [22,26,27]. Researchers in only a few studies over longer follow-up periods have analyzed the prognostic value of the method of tumor detection.

Most of the data derived from previous studies in which long-term outcomes of breast cancer detected within screening programs were analyzed are from clinical trials that reported mortality [28]. Only very few of the previous studies on screen detection and breast cancer prognosis focused on long-term survival of the patients (beyond 15 years from diagnosis) [3,29]. In these studies, the favorable survival of screen-detected cancers persisted over time. The data were adjusted for extent of disease, but not for a more detailed clinical, pathological or molecular profile.

There has been disagreement on the observed mortality and survival differences between patients whose breast cancer was detected by mammography and those whose breast cancer was detected outside screening, owing to biases in the statistical analysis [30-33]. In the context of patient survival, cancers found by screening mammography are detected earlier during their natural history and are therefore susceptible to lead time bias. In breast cancer, the lead time is estimated to be approximately 3 to 4 years [34]. The other main bias, duration bias, implies that slowly growing tumors are more likely to be detected by screening because they remain asymptomatic but detectable by mammography for a longer time [32,35].

Screen-detected cancers may be subject to selection bias in that true attendees may represent not the entire population, but rather a generally more health-conscious population. In addition, screening may lead to detection of indolent cancers that would never have caused symptomatic disease and therefore they are overdiagnosed.

In the present study, our aim was to evaluate whether the survival advantage of patients with screen-detected cancers persists after long-term follow-up. For this purpose, we analyzed an unselected nationwide series of breast cancer patients, which was recently updated to include long-term follow-up data. In previous studies of screen detection and survival, various definitions of screen-detected breast cancer have been used. In some studies, for example, outcomes of invited women and noninvited women have been compared [36], or interval cancers have been included in the screen-detected group [20,37], instead of the outcomes of true screening attendees with the outcomes of patients with cancer detected by other methods [36]. In the current study, we used hospital records to retrieve information about the method of detection and compared the survival of true attendees with the survival of patients with cancers detected outside screening. Survival estimates were adjusted according to an extensive series of factors known to reflect the extent of disease to reduce the effect of both lead time and length time biases. In addition, we analyzed the occurrence of contralateral breast cancer according to the method of tumor detection.

## Material and methods

### Patients

Five well-defined geographical areas comprising approximately 50% of the Finnish population were selected for the study. We identified 2,936 patients diagnosed with breast cancer in 1991 and 1992 from the files of the Finnish Cancer Registry, which constitutes 53% of all women diagnosed with breast cancer in Finland during that time period (*N *= 5,551). Permission to use clinical data and formalin-fixed, paraffin-embedded tissues for research purposes was provided by the Ministry of Social Affairs and Health, Finland (permission 123/08/97). With reference to the large number of studied cases, the authorities granted permission to use tissue samples without individual patient consent.

Clinicopathological data were extracted from the hospital records by using data collection forms, and tumor tissue samples from each patient's cancerous tissue were collected for tumor microarrays [38] as previously described in detail [9,17]. Outcome and cause of death data were compiled from the files of the Finnish Cancer Registry and Statistics Finland.

Of the 2,936 patients, 14 were excluded because of zero follow-up time due to perioperative mortality or diagnosis at autopsy. In 46 patients (2%), incorrect diagnoses were recorded during data extraction and thus were also excluded. The remaining 2,883 patients formed the FinProg database, which has previously been published and made available as an online case-match prognostic tool http://www.finprog.org/. Of the 2,883 patients, 131 (5%) had distant metastases at the time of diagnosis and were excluded. Patients who were not treated surgically (*n *= 101, 4%) and patients with lobular or ductal carcinoma *in situ *(*n *= 209, 7%) were excluded. Those with missing data on exclusion variables were excluded, except for missing metastasis status. All of the exclusion criteria mentioned above were used in subsequent analyses.

In patients with contralateral breast cancer or other cancer, the true origin of potential distant metastases may be difficult to determine. For the main analyses, patients with previous, synchronous or later contralateral invasive breast carcinoma were excluded (*n *= 349, 12%). Furthermore, we excluded patients with recent, synchronous or later other cancers (*n *= 301, 10%), except for cervical carcinoma *in situ *and basal cell carcinoma. We defined other cancer as recent if it had been diagnosed within 5 years before the index breast cancer. Thus all patients with other carcinoma diagnosed after 1 January 1985 were excluded. The total number of patients included in the main analyses was 1,934.

For the first sensitivity analyses, we excluded patients with contralateral breast cancer only if it had been diagnosed before or at the same time as the index breast cancer (*n *= 168, 6%). We also excluded patients with other carcinoma only if it was recent (1985 and later) or synchronous (*n *= 44, 2%). For the second sensitivity analyses, we included all patients with contralateral breast cancer or other cancer or both and used only the primary exclusion criteria mentioned above (M+, no surgery, ductal carcinoma *in situ *and lobular carcinoma *in situ*). In all analyses, one patient could have been excluded for several reasons. The consort diagrams of the study are given in Additional file 1. We used breast cancer-specific survival (BCSS) as an outcome measurement. The median follow-up time of surviving patients was 15.4 years.

### Method of detection

A screening mammography program was launched in Finland in 1987. During 1991 and 1992, Finnish municipalities were obligated by legislation to organize mammography screening programs for women 50 to 59 years of age. A few of the municipalities, however, screened other age cohorts as well (women 40 to 49 years and/or older than 60). The information regarding the method of detection in the FinProg series was collected from hospital records and from the Mass Screening Registry, which is a department of the Finnish Cancer Registry. Consequently, in the FinProg database, patients diagnosed within the mammography screening program are patients who have been invited to have and also truly attended screening mammography.

For cancers diagnosed on the basis of symptoms, as interval cancers between screening rounds or for any other reason outside the mammography screening program, we use the term "detected outside screening" or "non-screen-detected breast cancer." Screening round was not considered in this study.

### Histopathological characteristics

Postsurgical tumor size was recorded according to information extracted from hospital records. Tumor measurements were done in the following order of priority: on tumor slides by a pathologist, at the time of surgery by a surgeon, on the basis of x-ray mammography by a radiologist or by clinical palpation. The largest tumor diameter was recorded as its postsurgical size. In the main analyses, there were 1,262 (65%) breast cancers whose tumor measurements were done by the pathologist on the basis of tumor slides. Similar distributions of methods of measurement were seen in both screen-detected and non-screen-detected breast cancers, where 267 (65%) and 974 (66%) tumor measurements, respectively, were done by a pathologist and 86 (21%) and 301 (20%), respectively, were done by a surgeon. More tumors were screen-detected when measured by a radiologist (*n *= 32, 8%) than when diagnosed by other methods (*n *= 68, 5%). Palpation as a method of measurement was used in 5 patients (1%) diagnosed by screening mammography compared to 51 patients (4%) diagnosed on the basis of other methods (Additional file 2, Table S1). Histological typing and evaluation of the grade components (mitotic cell count, nuclear pleomorphism and tubule formation) were usually performed according to the World Health Organization classification [39], although the criteria used in tumor classification cannot be stated with certainty in retrospect. The tumors were classified into three histological types: ductal carcinoma (not otherwise specified, including apocrine, mixed mucinous and atypical medullary types), lobular carcinoma (infiltrating lobular carcinoma with variants) and the special histological types (tubular, medullary, cribriform, papillary and pure mucinous carcinomas).

### Laboratory methods

Immunohistochemical (IHC) staining, evaluation of protein expression and *in situ *hybridization were performed as described in detail elsewhere [9,17,40,41]. According to recent recommendations by the American Society of Clinical Oncology, immunostaining of hormone receptors was considered positive when at least 1% of the cancer cells showed staining and negative when less than 1% of the cancer cells were stained [42]. For the other biomarkers, IHC expression was considered negative when less than 10% of tumor cell nuclei expressed protein, except for Ki-67 and p53, for which we used a cutoff of 20%.

### Statistical analysis

Frequency tables were analyzed using the χ^2 ^test. Life tables were calculated according to the Kaplan-Meier method. BCSS was computed from the date of the diagnosis until death due to breast cancer. The logrank test was used to compare survival between subgroups. Multivariate survival analyses were performed using a Cox proportional hazards model by entering the following covariates: method of tumor detection (mammography screening = 0, outside screening = 1), grade (well-differentiated = 0, moderately or poorly differentiated = 1), ER and progesterone receptor (PR) status (positive = 0, negative = 1), histological type (lobular or special = 0, ductal = 1), and age at detection grouped to account for the nonlinear risk associated with age. The tumor size in centimeters and the number of metastatic axillary lymph nodes were entered into the multivariate model as continuous variables. Multivariate analysis of the risk of later contralateral breast cancer was performed using logistic regression by entering the same covariates into the model as were entered into the Cox proportional hazards model. A *P-*value of 0.05 was adopted as the limit for inclusion of a covariate. All *P*-values are two-sided. STATA version 10 statistical software (StataCorp, College Station, TX, USA) was used for the analyses.

## Results

### Method of tumor detection and clinicopathological features

In the main study series (*N *= 1,884), 408 patients (22%) had screen-detected breast cancer and 1,476 (78%) had non-screen-detected breast cancer. Mammography screening is organized mainly for women 50 to 69 years of age. In the current series, 484 patients (26%) were ages 50 to 59 years and 365 (19%) were ages 60 to 69 years. Of these patients, 254 (52%) and 93 (25%) were patients with screen-detected cancer, respectively, and 230 (48%) and 272 (75%) had non-screen-detected cancer, respectively.

The median tumor size for the entire cohort was 20 mm. The median tumor size was 13 mm among screen-detected tumors, and it was 20 mm in non-screen-detected tumors. Eighty-three percent of screen-detected tumors were 20 mm or less (T1), compared to only 53% of non-screen-detected tumors.

Nodal involvement was less frequent in patients with screen-detected breast cancer than in non-screen-detected breast cancer (21% vs 37%; *P *< 0.001), which was also true among women 50 to 69 years of age (20% vs 35%; *P *< 0.001). Among the screen-detected cancers, 66% of tumors were 20 mm or less and had no nodal involvement (T1N0), and only 36% of non-screen-detected cancers belonged to this category.

The majority of screen-detected tumors were ductal carcinomas (70%). However, the proportion of ductal carcinomas was significantly higher (75%; *P *< 0.04) in non-screen-detected cancers. The proportion of lobular carcinomas (16%) was equal in both diagnostic groups. Screen-detected tumors were more often of a special histological type (14% vs 9%; *P *< 0.04). Similar differences in histological profile were seen in the 50- to 69-year-old age cohort (ductal: 70% vs 73%; lobular: 16% vs 19%; special type: 14% vs 8%; *P *= 0.02).

Tumors detected by mammography screening were more often of a lower grade of differentiation. There were 129 grade 1 breast cancers (32%) in the screen-detected group and 241 (16%) among tumors detected by other methods (*P *< 0.001). Correspondingly, screen-detected cancers were less often grade 2 or 3 tumors (*n *= 202, 49%) than were non-screen-detected cancers (*n *= 841, 57%) (*P *< 0.001). However, this association weakened with increasing tumor size (Additional file 2, Table S2). In T1N0 tumors, 36% of screen-detected tumors were well-differentiated, but only 22% in the non-screen-detected group were. In patients with larger tumors, such as T2 tumors (21 to 50 mm), the proportion of G1 tumors was equal in both diagnostic groups (11%) and the proportion of G2-3 tumors was 75% in screen-detected tumors compared to 64% in non-screen-detected tumors (*P *= 0.77).

Screen detection was associated with hormone receptor status (ER and PR), although in analyses consisting of all age groups, the association was weak (*P *= 0.038 and *P *= 0.094, respectively). In women at screening age (50 to 69 years), there were only 9% ER- tumors in screen-detected tumors compared to 19% in non-screen-detected tumors (*P *= 0.002). Similar differences were seen according to PR status (*P *< 0.001).

No statistically significant association between screen detection and *HER2 *gene amplification or p53 expression was seen in any of the analyzed age groups. Somewhat fewer Ki-67-positive tumors were seen in screen-detected breast cancers compared to those detected outside screening (18% vs 26%), but the association was not statistically significant (*P *= 0.08).

Adjuvant systemic therapy was given less frequently to patients with screen-detected breast cancer than to women with non-screen-detected breast cancer (22% vs 41%; *P *< 0.001). The use of adjuvant systemic therapy was not known in 5 patients (1%) with screen-detected cancers and in 40 patients (3%) with non-screen-detected cancers.

In women 50 to 69 years of age, screen detection was statistically significantly associated with smaller primary tumor size, less frequent lymph node metastasis, special histological type, lower grade of differentiation and positive hormone receptor status (ER and PR) compared with non-screen-detected cancers. The distribution of clinicopathological features in women with breast cancer diagnosed by screening mammography and by other methods is shown in Table 1.

**Table 1:** Descriptive statistics for main analyses (*N *= 1,884)

	All age groups			Ages 50 to 69 years		
	
Factor	**Screening, *n *(%)**^**a**^ (*n *= 408)	Outside screening, *n *(%) (*n *= 1,476)	*P *value	Screening, *n *(%) (*n *= 347)	Outside screening, *n *(%) (*n *= 502)	*P *value
Primary tumor diameter (mm)			< 0.001			< 0.001
≤ 10	158 (39)	207 (14)		143 (41)	82 (16)	
11 to 20	179 (44)	576 (39)		147 (42)	204 (41)	
21 to 50	55 (13)	581 (39)		42 (12)	183 (36)	
> 50	2 (0)	66 (4)		1 (0)	21 (4)	
N.A.	14 (3)	46 (3)		14 (4)	12 (2)	
Nodal status			< 0.001			< 0.001
Negative	317 (77)	862 (58)		273 (79)	321 (64)	
Positive	85 (21)	552 (37)		69 (20)	178 (35)	
N.A.	6 (1)	62 (4)		5 (1)	3 (0)	
Histological type			0.04			0.02
Ductal	287 (70)	1,104 (75)		243 (70)	365 (73)	
Lobular	65 (16)	232 (16)		55 (16)	95 (19)	
Special type	56 (14)	139 (9)		49 (14)	41 (8)	
N.A.	0	1 (0)		0	1 (0)	
Histological grade			< 0.001			< 0.001
1	129 (32)	241 (16)		111 (32)	85 (17)	
2	147 (36)	515 (35)		123 (35)	172 (34)	
3	55 (13)	326 (22)		43 (12)	115 (23)	
N.A.	77 (19)	394 (27)		70 (20)	130 (26)	
ER content			0.038			0.002
Negative	40 (10)	232 (16)		31 (9)	94 (19)	
Positive	204 (50)	802 (54)		172 (50)	260 (52)	
N.A.	164 (40)	442 (30)		144 (41)	148 (29)	
PR content			0.094			< 0.001
Negative	79 (19)	392 (27)		64 (18)	171 (34)	
Positive	161 (39)	620 (42)		132 (38)	173 (34)	
N.A.	168 (41)	464 (31)		151 (44)	158 (31)	
HER2						
Amplification			0.09			0.19
Negative	217 (53)	852 (58)		176 (51)	293 (58)	
Positive	37 (9)	202 (14)		32 (9)	72 (14)	
N.A.	154 (38)	422 (29)		139 (40)	137 (27)	
Expression			0.25			0.33
Negative	218 (53)	889 (60)		179 (52)	299 (60)	
Positive	39 (10)	198 (13)		33 (10)	69 (14)	
N.A.	151 (37)	389 (26)		135 (39)	134 (27)	
Ki-67			0.08			0.07
Negative	155 (38)	615 (42)		133 (38)	209 (42)	
Positive	73 (18)	379 (26)		54 (16)	121 (24)	
N.A.	180 (44)	482 (33)		160 (46)	172 (34)	
p53			0.63			0.21
Negative	171 (42)	786 (53)		145 (42)	254 (51)	
Positive	38 (9)	192 (13)		31 (9)	73 (15)	
N.A.	199 (49)	498 (34)		171 (49)	175 (35)	
Adjuvant systemic therapy			< 0.001			< 0.001
Not given	314 (77)	827 (56)		271 (78)	297 (59)	
Given	89 (22)	609 (41)		72 (21)	197 (39)	
N.A.	5 (1)	40 (3)		4 (1)	8 (2)	

ER = estrogen receptor; PR = progesterone receptor; N.A. = not available. ^a^Percentages may not equal 100% due to rounding.

### Method of detection and breast cancer survival

**Figure 1:**
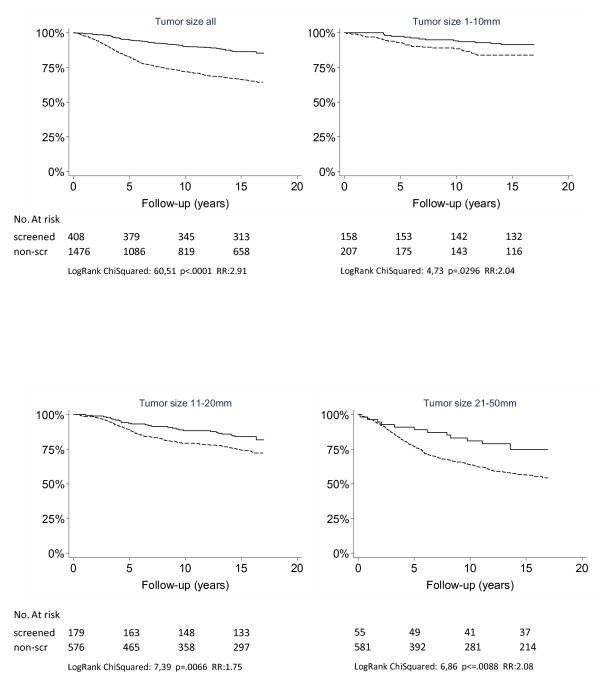
**Breast cancer-specific survival by primary tumor size and mode of detection**. Solid lines represent cancers detected by mammography screening, dashed lines represent cancers detected outside screening.

Women with screen-detected breast cancer had more favorable BCSS than patients with non-screen-detected cancer. The 15-year BCSS rate for patients with screen-detected breast cancer was 86%, and for patients with non-screen-detected breast cancer it was 66% (*P *< 0.0001, RR = 2.91, RR = risk ratio). The survival advantage was seen in tumor size categories 1 to 10 mm, 11 to 20 mm and 21 to 50 mm (Figure 1). Only two patients available for analysis in the screen-detected group had tumors larger than 50 mm, which did not allow a statistical comparison. Women with screen-detected T2 category tumors (21 to 50 mm) had prognoses similar to those of patients with non-screen-detected T1 category tumors (1 to 20 mm) (15-year BCSS 76.6% vs 77%; *P *= 0.863 and RR = 0.95) (Table 2).

**Table 2:** Breast cancer-specific survival according to primary tumor diameter

	Screening		Outside screening			
			
Primary tumor diameter (mm)	At risk (*n*)	15-year survival (%)	At risk (*n*)	15-year survival (%)	*P *value	RR
All age groups						
≤ 10	132	92.1	116	83.9	0.0296	2.04
11 to 20	133	84	297	74.5	0.0066	1.75
21 to 50	37	76.6	214	56.5	0.0088	2.08
> 50	N.A.	N.A.	19	34.1	N.A.	N.A.
Ages 50 to 69 years						
≤ 10	122	92.1	54	83.1	0.0343	2.25
11 to 20	110	81.5	123	74.6	0.0764	1.52
21 to 50	30	77.7	89	59.4	0.0325	1.02
> 50	N.A.	N.A.	5	35.7	N.A.	N.A.
Node-negative ages 50 to 69 years						
≤ 10	114	93.7	48	84	0.021	2.66
11 to 20	84	87	89	79.3	0.0534	1.85
21 to 50	19	90.7	58	70.9	0.0769	2.79
> 50	N.A.	N.A.	2	50	N.A.	N.A.

N.A. = data not available because screen-detected tumors were rarely > 50 mm; RR = risk ratio.

### Influence of screening on outcome according to nodal status

This study included 1,203 (62%) node-negative and 654 (34%) node-positive breast cancer patients. Among the node-negative breast cancer patients, breast cancer was screen-detected in 317 patients (78%) and non-screen-detected in 862 patients (58%) (*P *< 0.001). In patients with screen-detected node-negative breast cancer, 15-year BCSS was 91% compared to 77% in the non-screen-detected group (*P *< 0.0001 and RR = 3). Patients with screen-detected, node-positive breast cancer had a survival advantage compared to patients diagnosed by other methods (71% vs 51%, respectively; *P *= 0.0011 and RR = 1.95).

### Influence of age at diagnosis on outcome

In this study, there were 873 patients (45%) ages 50 to 69 years, of whom 347 (40%) were diagnosed by screening and 502 (58%) were diagnosed on the basis of other methods. There was a statistically significant difference in 15-year BCSS between the two subgroups (15-year BCSS 86% for patients with screen-detected tumors and 68% for patients diagnosed by other methods; *P *< 0.0001 and RR = 2.61). Similar differences in survival were observed in all other age cohorts (Table 3).

**Table 3:** Breast cancer-specific survival by method of detection and age at diagnosis

	Screening				Outside screening			
	
		Survival (%)				Survival (%)		
Age at diagnosis (years)	Patients (*n*)	5 years	10 years	15 years	Patients (*n*)	5 years	10 years	15 years
Node-negative and node-positive								
≤ 39	2	N.A.	N.A.	N.A.	118	81.4	63.5	57.3
40 to 49	47	100	93.5	93.5	362	85.6	75.8	71.3
50 to 59	254	94.1	89.6	86.3	230	84.2	77.1	71.5
60 to 69	93	97.8	92.1	84.9	272	83.3	71.2	65.8
≥ 70	12	N.A.	N.A.	N.A.	494	80.4	69.1	60.8
Node-negative								
≤ 39	1	N.A.	N.A.	N.A.	64	92.2	81.2	76.3
40 to 49	34	100	97.1	97.1	195	92.8	85.6	82.5
50 to 59	190	98.4	95.2	92.4	150	92	86.5	82.3
60 to 69	83	98.9	92.5	87.2	171	86.1	79.1	72.1
≥ 70	9	N.A.	N.A.	N.A.	282	88.6	78	70.7
Node-positive								
≤ 39	1	N.A.	N.A.	N.A.	54	72.2	46.3	38.6
40 to 49	12	100	91.7	91.7	165	76.9	64	57.8
50 to 59	61	83.4	75	69.9	79	69.5	59.2	50.9
60 to 69	8	100	100	71.4	99	78.4	58.4	55.6
≥ 70	3	N.A.	N.A.	N.A.	155	66	55.5	45.4

N.A. = data not available because screening was rarely performed in women ages ≤ 39 and ≥ 70.

### Multivariate survival analyses

Screening mammography is commonly offered to women 50 to 69 years of age. Because the proportion of screen-detected cancers was different according to age groups, and since screen-detected tumors were smaller, less often node-positive, less often of the ductal type, more differentiated, and associated with a more favorable hormonal status, we performed a multivariate analysis to adjust for these factors. As a result, the method of tumor detection was an independent prognostic factor, with a hazard ratio (HR) of 1.69 (95% CI = 1.06 to 2.70) between patients whose tumors were detected outside screening and those whose tumors were screen-detected. Tumor size, nodal status, tumor grade and *HER2 *amplification OK were also independent of other variables (Table 4). The HR for the method of detection was 1.48 (95% CI = 0.97 to 2.25) in the first sensitivity analysis and 1.45 (95% CI = 0.97 to 2.17) in the second sensitivity analysis.

**Table 4:** Cox multivariable analysis

Variable	HR (95% CI)	*P *value
Detection outside screening	1.69 (1.06 to 2.70)	0.028
Tumor size (per cm)	1.02 (1.01 to 1.03)	< 0.001
Positive lymph nodes (*n*/metastatic node)	1.31 (1.10 to 1.17)	< 0.001
Histological grade (grade II vs grade III vs grade I)	2.54 (1.52 to 4.25)	< 0.001
PR (negative vs positive)	1.23 (0.92 to 1.64)	0.154
HER2 amplification (positive vs negative)	1.45 (1.06 to 1.97)	0.019
Age at diagnosis (years)		
≤ 39^a^	1.00	
40 to 49	0.62 (0.28 to 1.00)	0.051
50 to 59	0.82 (0.50 to 1.34)	0.437
60 to 69	0.92 (0.57 to 1.49)	0.733
≥ 70	1.13 (0.70 to 1.83)	0.605

^a^Reference category.

### Contralateral breast cancer

Differences in the probability of having future contralateral breast cancer between screen-detected and non-screen-detected breast cancer patients during the 15-year follow-up time were assessed. For this analysis, we used the same exclusion criteria that we applied in the main analysis, with the exception of later contralateral breast cancers. Without any adjustments according to other variables, there were no significant differences if the primary breast cancer was diagnosed by screening mammography or by other methods (7.3% vs 6.8%, respectively; *P *= 0.74 and OR = 0.96). When patients 50 to 69 years of age were analyzed, the results were similar (7.0% vs 7.9%, respectively; *P *= 0.60, respectively). After adjustments according to the same covariates as those used in the Cox multivariate survival analyses, the risk of having later contralateral breast cancer was slightly increased in patients with breast cancer detected outside screening compared to patients with screen-detected breast cancer (95% CI = 0.55 to 2.57 and OR = 1.18), but the difference was not statistically significant.

## Discussion

In the current study, we have shown that the outcome of breast cancer is significantly better in patients with mammography screen-detected tumors than in patients with tumors detected by other methods, even beyond 15 years after diagnosis. After adjustment for a series of potential confounders, patients whose tumors were detected during screening mammography had an approximately 41% lower risk of dying as a result of breast cancer than did those whose tumors were detected outside screening. These results are in accord with those of our previous studies [9,17] in which we found significant survival differences over postdiagnostic follow-up durations up to 10 years.

The survival difference could not be explained by lead time- and length bias-related variables, such as extent of disease, histopathological and molecular prognostic factors. Similar results have been reported by others [3,18,19,29] who have shown the method of detection to be an independent prognostic factor. An adjusted risk decrease ranging from 21% to 48% for patients detected within screening has been reported, which is similar to the effect size found in the current study [3,18,19].

In agreement with previous studies, we found that screen-detected tumors were smaller and were less frequently node-positive than tumors detected outside screening. Screen-detected tumors were more often well-differentiated, of special histological type, hormone receptor-positive and showed lower proliferation as measured by Ki-67 expression. The only examined biomarker that was not significantly associated with the method of detection was *HER2 *amplification. A similar finding has previously been reported for *HER2 *expression [2,6].

The adjusted HR (1.69) between patients with screen-detected and non-screen-detected cancers according to BCSS was somewhat lower, with a median follow-up of 15.4 years compared to the HR (2.1) after 9.5 years follow-up in our previous study [17]. This finding could potentially be explained by a large proportion of patients who were excluded (contralateral breast cancer or other cancer) during the extended follow-up in the main analyses. It is not supported by the sensitivity analyses, however, because the HR was lower (1.47) when patients with contralateral cancers were included.

The risk of contralateral breast cancer among breast cancer survivors is reportedly two- to sixfold the risk of breast cancer in the general population [43]. The risk of having contralateral breast cancer among breast cancer survivors is associated with lobular or inflammatory histology of the primary breast cancer, family history of breast cancer and young age at the time of primary diagnosis [44-47]. To the best of our knowledge, the association between the method of detection of primary cancer and the risk of contralateral breast cancer has not been investigated previously. In current study, we found that the risk for contralateral breast cancer was similar in patients with screen-detected and non-screen-detected cancers. However, the risk of contralateral breast cancer is reported to be significantly higher among women with primary *in situ *cancer [48]. The proportion of *in situ *cancers is higher in screen-detected than in non-screen-detected cancers. Only patients with invasive breast cancers were included in the current study. Whether the risk of contralateral breast cancer is altered according to the method of detection in women with primary *in situ *cancer remains to be investigated. According to this study, the follow-up for contralateral breast cancer should be similar in patients with screen-detected and non-screen-detected invasive breast cancers.

In the current study, screen-detected cancer was defined according to true attendance at mammography screening. All other breast cancers, including those of patients who did not undergo mammography, were considered non-screen-detected. If we had included nonattendees in the screen-detected group, the survival estimates could be distorted, thus underestimating the survival advantage of screening. In a previous study, breast cancer mortality among the women invited to have mammographies was reduced by 22%, whereas the mortality reduction was 28% among true attendees [49].

The use of adjuvant therapies was rather homogeneous during 1991 and 1992 in Finland. In this cohort, adjuvant therapy was given less frequently to patients with screen-detected tumors than to patients with breast cancer diagnosed by other methods. Thus the use of adjuvant therapies does not explain the survival advantage associated with screening in this study.

Although there were significant differences in the proportion of many commonly used prognostic factors between screen-detected and non-screen-detected tumors, the frequency of lobular carcinoma was similar in both groups. Lobular carcinomas are often difficult to detect by mammography because they are radiologically elusive [50,51]. Lobular carcinomas may also be difficult to detect clinically because they less commonly form a palpable mass [52], which may in part explain the similar proportion of lobular carcinomas in both groups.

Although we were able to adjust the HRs for the method of detection for an extensive series of known prognostic factors, survival differences remain to be explained. More detailed tumor profiling might aid in characterizing tumors and should be studied in future projects. The value of some commonly used prognostic factors is observed to diminish over prolonged follow-up times. In the present study, the screening benefit remained after long-term follow-up. According to this study, patients with screen-detected breast cancer may be overtreated if screening as a method of detection is not taken into account in risk estimation and therapy decision-making.

## Conclusions

The method of detection is an independent prognostic factor for long-term survival among breast cancer patients. According to this study, screening as a method of detection may be considered a favorable prognostic factor in risk estimation and therapy decision-making to avoid overtreatment of screen-detected breast cancers.

## Abbreviations

CISH: chromogenic *in situ *hybridization; ER: estrogen receptor; HER2: human epidermal growth factor receptor 2; PR: progesterone receptor.

## Competing interests

The authors declare that they have no competing interests.

## Authors' contributions

TL, HJ and JL designed the study and drafted the manuscript. HJ initiated the FinProg study and organized the collection of clinical data. JL, ML, TL, KH, LE, VK, TTH and JI collected the clinical data. JI organized the collection of tumor samples. TL collected the tissue for tissue microarrays. JI, HS and NL interpreted the results of the immunohistochemical and *in situ *hybridization analysis. TL and JL performed the statistical analyses. All authors critically revised the manuscript and approved its final form.

## Supplementary Material

Additional file 1 **Consort diagrams for main analyses, the first sensitivity analyses and the second sensitivity analyses**. Click here for file

Additional file 2 **Table S1 Distribution of breast cancers in the FinProg data according to the method of measurement and the method of detection (exclusions according to main analyses, *N *= 1,884)**. Table S2 Adjustment according to tumor size after analyzing proportion of different grades in screen-detected and non-screen-detected patients (exclusions according to main analyses, *N *= 1,884). Click here for file
