# Supplementary material for: Long-term prognosis of breast cancer detected by mammography screening or other methods
Source: Breast Cancer Res. 2011 Dec 28;13(6):R134. doi: 10.1186/bcr3080 (PMC3326576; doi:10.1186/bcr3080)
Supplement: Additional file 2 — Table S1 Distribution of breast cancers in the FinProg data according to the method of measurement and the method of detection (exclusions according to main analyses, N = 1,884). Table S2 Adjustment according to tumor size after analyzing proportion of different grades in screen-detected and non-screen-detected patients (exclusions according to main analyses, N = 1,884). [file bcr3080-S2.DOCX]

Additional file 2, Table 1.

|  | | | | | | | | | | |
| --- | --- | --- | --- | --- | --- | --- | --- | --- | --- | --- |
|  |  |  |  |  |  |  |  |  |  |  |
|  | | | | |  |  |  |  |  |  |
|  |  |  |  |  |  |  |  |  |  |  |
|  |  | **all** |  | **screened** | | **not screened** | |  |  |  |
|  |  |  |  | **(of all screened cases =408)** | | **(of all not screened cases =1,476)** | | | |  |
|  |  |  |  |  |  |  |  |  |  |  |
| patologist |  | 1262 (65%) | | 267 (65%) |  | 974 (66%) |  |  |  |  |
| surgeon |  | 388 (20%) |  | 86 (21%) |  | 301 (20%) |  |  |  |  |
| radiologist | | 100 (5%) |  | 32 (8%) |  | 68 (5%) |  |  |  |  |
| palpation |  | 59 (3%) |  | 5 (1%) |  | 53 (4%) |  |  |  |  |
| other method | | 22 (1%) |  | 3 (1%) |  | 19 (1%) |  |  |  |  |
| not avalilable | | 103 (5%) |  | 15 (4%) |  | 61 (4%) |  |  |  |  |
|  |  |  |  |  |  |  |  |  |  |  |
| Percentages may not equal 100 due to rounding | | | | |  |  |  |  |  |  |

| Additional file 2, Table 2. | | | | | |  |
| --- | --- | --- | --- | --- | --- | --- |
|  | | |  |  |  |  |
|  |  |  |  |  |  |  |
|  | screened ≤10mm | not screened ≤10mm | screened 11-20mm | not screened 11-20mm | screened 21-50mm | not screened 21-50mm |
| total | 158 | 207 | 179 | 576 | 55 | 581 |
| G1 | 77 (49%) | 66 (32%) | 43 (24%) | 106 (18%) | 6 (11%) | 62 (11%) |
| G2-3 | 57 (36%) | 81 (39%) | 97 (54%) | 320 (56%) | 41 (75%) | 370 (64%) |
|  |  |  |  |  |  |  |
| Percentages do not equal 100: grade was not always available for analysis | | | |  |  |  |

p=0.035 p=0.174 p=0.768
